# Sildenafil and risk of Alzheimer disease: a systematic review and meta-analysis

**DOI:** 10.18632/aging.206222

**Published:** 2025-03-17

**Authors:** Wei Yu Chua, Lincoln Kai En Lim, James Jia Dong Wang, Aaron Shengting Mai, Ling-Ling Chan, Eng-King Tan

**Affiliations:** 1 Yong Loo Lin School of Medicine, National University of Singapore, Singapore, Singapore; 2 Lee Kong Chian School of Medicine, Nanyang Technological University, Singapore, Singapore; 3 Department of Diagnostic Radiology, Singapore General Hospital Campus, National Neuroscience Institute, Singapore, Singapore; 4 Department of Neurology, Singapore General Hospital Campus, National Neuroscience Institute, Singapore, Singapore; 5 Neuroscience and Behavioral Disorders, Duke-NUS Medical School, Singapore, Singapore

**Keywords:** Alzheimer disease, Sildenafil, meta-analysis, PDE5 inhibitors

## Abstract

Background: Alzheimer Disease (AD) affects more than 50 million people worldwide, with 10 million new diagnosis each year. The link between Sildenafil, a Phosphodiesterase-5 (PDE5) inhibitor, and risk of AD has been debated. We conducted the first meta-analysis on the association between Sildenafil use and risk of AD.

Methods: We searched MEDLINE and Embase from inception to March 11, 2024 to identify cohort, case-control studies comparing the frequency of AD in patients taking Sildenafil with those without. We computed risk ratios (RR) and hazard ratios (HR) with accompanying 95% Confidence Intervals (CIs) for each study, and pooled the results using a random-effects meta-analysis.

Results: Out of 415 studies that were screened initially, 5 studies comprising 885,380 patients were included for analysis. Sildenafil use was associated with a reduced risk of developing AD by two-fold compared to non-use (HR: 0.47, 95% CI: 0.27-0.82, p<0.001). There was a similar association in risk reduction of AD in patients on PDE5 inhibitors compared to non-use (RR: 0.55, 95% CI: 0.38-0.80, p=0.002).

Conclusions: Our meta-analysis showed that the use of Sildenafil is associated with a reduced risk of developing AD by two-fold. Further randomized control trials to ascertain the effect of Sildenafil on AD pathology would be useful.

## INTRODUCTION

Alzheimer Disease (AD) is a highly debilitating neurogenerative disease that affects more than 50 million people worldwide, with 10 million new cases diagnosed each year. Among adults older than 65 years old, AD remains the fifth leading cause of death in America, with estimated total healthcare costs exceeding USD $300 billion annually [1]. As the proportion of elderly people continue to increase, the cost of AD is expected to exceed USD $1 trillion with significant implication on healthcare systems and caregivers [2]. The impact of AD is far reaching, with secondary effects on caregivers, employment opportunities and society [3]. Therefore, it is imperative to manage and reduce the development of AD in at risk patients.

While the World Health Organization (WHO) designated AD as a public health priority, there are presently no definitive treatments [4]. Many therapeutic drugs have been trialled in patients with AD, [5, 6] and the current management strategy involves the prevention of AD development through pharmacological and non-pharmacological therapies as well as symptomatic management [7].

Sildenafil (under brand name Viagra and others), a Phosphodiesterase-5 (PDE5) inhibitor, was approved for medical use since 1998 and has more than 3 million prescriptions in United States alone in 2021 [8]. In animal studies, the use of PDE5 inhibitors improves the learning and memory of mouse models [9]. Among the PDE5 inhibitors, Sildenafil has shown to reduce the levels of amyloid-β (Aβ) peptide, a hallmark of AD, in the hippocampus of mouse models. Multiple pathways linked to the activity of Sildenafil have been implicated and the nitric oxide synthase/nitric oxide/cyclic guanosine monophosphate (NOS/NO/cGMP) signalling pathway has been demonstrated to be key in slowing the progression of AD.

However, studies in human population have been limited and the relationship between Sildenafil and risk of AD has been debated. A case control study by Fang et al. found that the use of Sildenafil was associated with reduced risk of AD compared to non-use (HR: 0.31, 95% CI: 0.25-0.39) while Desai et al. found that the use of PDE5 inhibitors was not associated with reduced risk of AD [10, 11]. However, a recent large cohort study by Adesuyan et al. supported the protective effect of Sildenafil in patients with AD (HR: 0.81, 95% CI: 0.71-0.93) [12].

To date, there has been no meta-analysis to examine the association between use of Sildenafil and AD risk. To address this gap in knowledge, we conducted a systematic review and meta-analysis to explore the following outcomes: (1) the use of Sildenafil in reducing the risk of AD (2) and the use of PDE5 inhibitors in reducing the risk of AD.

## MATERIALS AND METHODS

This meta-analysis was registered with PROSPERO at CRD42024524114 and conducted in accordance to the reporting guidelines of Preferred Reporting Items for Systematic Reviews and Meta-analyses (PRISMA) [13].

### Information source and search strategy

A systematic search was conducted MEDLINE and Embase using Medical Subject Headings (MeSH) and keywords. Keywords and MeSH terms synonymous with “Sildenafil”, “Phosphodiesterase-5 inhibitor” and “Alzheimer Disease” formed the basis of the search strategy. The search period includes articles from inception to March 11, 2024. Only full text articles published in the English language were included. The full search strategy and search terms are included in Supplementary Table 1. References were imported into EndNoteX9 for the initial removal of duplicates.

### Study selection

Two authors (W.Y.C. and L.K.E.L.) reviewed each reference in a blinded manner and any disagreements were resolved through discussion or referred to a third independent author for the final decision (A.S.M.). The review was carried out in 2 stages: first, the titles and abstracts were reviewed and second, the full texts of selected references were retrieved and reviewed. Original studies, published in English, discussing Sildenafil in adults with AD were included. Accepted study designs included case control and cohort studies. We excluded non-peer reviewed articles, review articles (including other systematic reviews and meta-analyses), editorials, letters to editor, and conference abstracts. Studies involving animal or non-human studies were also excluded.

### Data extraction

Two investigators (W.Y.C. and J.J.D.W.) independently extracted information from the included studies. The data collected included authors, year of publication, total number of participants, age and sex of study participants, sample size, type of treatment, and AD outcome. Regarding discrepancies, a third author (L.K.E.L.) was consulted to make the final decision regarding the data extraction process.

### Quality assessment

The Newcastle-Ottawa Quality Assessment Scale was used to assess the risk of bias of the included studies [14]. Two investigators (L.K.E.L. and J.J.D.W.) independently reviewed all included studies and rated them based on the following domains: selection of study group, comparability of selected groups, and measurement of outcome of interest. Subsequently, gradings for each domain were compared between the two authors and in case of disagreements, a third independent author (W.Y.C.) was consulted, and a consensus was reached through discussion. The Newcastle-Ottawa scale was subsequently converted to Agency for Healthcare Research and Quality (AHRQ) standards (good, fair or poor quality studies). A study with ≥7 points was considered as “good”, 2 to 6 points were considered as “fair”, and ≤1 point was considered as “poor” quality.

### Data analysis

All analyses were undertaken using Review Manager 5.4.1 and RStudio version 4.3.3. The statistical packages used in R were (tidyverse; meta; metafor; ggplot2; gridExtra and dmetar) [15]. The random effects model was used to estimate the pooled risk ratios and its corresponding 95% confidence intervals (CIs). A risk ratio <1 indicates that Sildenafil is associated with a lower risk of AD. Data from Fang et al., Desai et al., Huo et al., Braun et al. and Adesuyan et al. were used to calculate Risk Ratios (RRs) [10–12, 16, 17]. The proportion of variability due to heterogeneity was assessed using the I^2^ statistic. We considered heterogeneity to be significant when the I^2^ statistic was ≥25%. In cases with high heterogeneity, a random-effects model was used. Precalculated log-transformed hazards ratios (HRs) were pooled using the inverse variance method [15]. Fang et al., Huo et al. and Adesuyan et al. were utilized to calculate Hazard Ratios (HRs) [11, 12, 17]. The level of significance is defined as p <0.05. All results were presented as their effect sizes with the accompanying 95% CIs, along with the p-values where applicable.

For meta-analyses that have high heterogeneity, we performed an influence analysis to determine the contribution of each study to the overall heterogeneity. Based on the resultant Baujat plots and leave-one-out analyses, as well as inspection of the forest plots, we performed a sensitivity analysis in which outliers were excluded [15].

### Availability of data and materials

The datasets used and/or analysed during the current study are available from the corresponding author on reasonable request.

## RESULTS

### Overview

**Figure 1:**
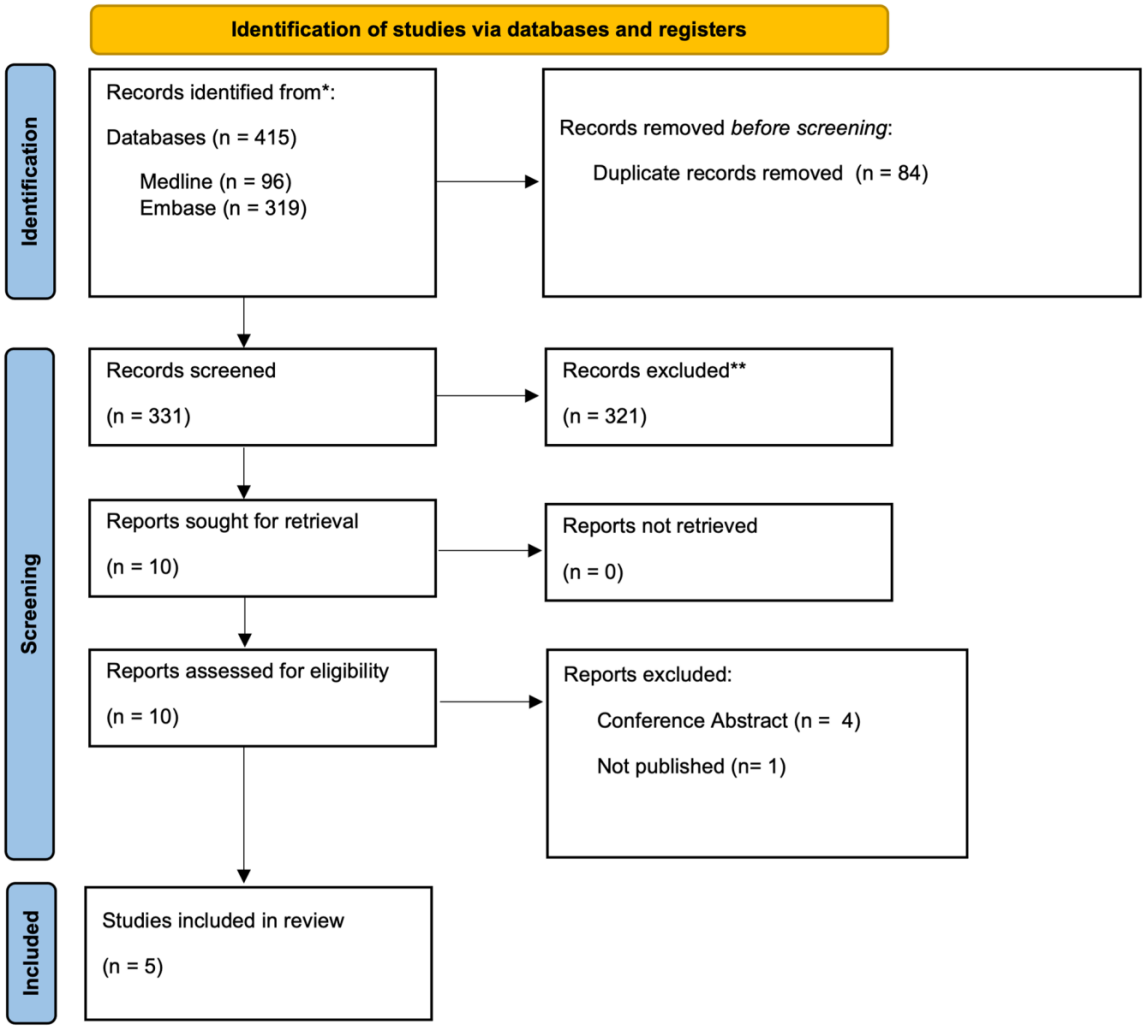
PRISMA flowchart.

A total of 415 studies were found after searching MEDLINE and Embase. Among these, 84 were duplicates and 331 studies remained following duplicate removal. The study team screened the titles and abstracts of these studies and included 10 studies for further review. The study team retrieved the full texts of these 10 studies and 5 studies involving 885,380 patients were included in the final analysis [10–12, 16, 17] (Figure 1).

### Characteristics of included studies

The 5 studies included 3 cohort studies [10, 12, 16] and 2 case control studies [11, 17]. The treatment arm varies between the 5 studies. Three studies compared the efficacy of Sildenafil only, [11, 16, 17] one study compared the efficacy of PDE5 inhibitors (Sildenafil or Tadalafil), [10] and the remaining one study compared the efficacy of PDE5 inhibitors (Sildenafil, Tadalafil or Vardenafil) [12]. A summary of the characteristics of included studies can be found in Table 1.

**Table 1:** Summary of included studies.

**Author, year**	**Study design**	**Study setting/ database**	**Inclusion criteria**	**Treatment**	**Control**	**Sample size**	**Female, No. (%)**	**Age, y** **mean, SD/** **median, (IQR)**	**Alzheimer dementia, no.**	**Median (IQR) duration of follow up**
Fang, 2021^a^	Retrospective Case Control	USA: MarketScan Medicare Claims database (2012-2017)	(1) Only first sildenafil episode	Sildenafil	Non-use	Total: 576,768 Treatment: 116,412 Control: 460,356	11535 (2.0%)	73.6 (7.1)	Total: 1268 Treatment: 93 Control: 1,175	Treatment: 6 years Control: 6 years
Desai, 2022^b^	Retrospective Cohort Study	USA: Medicare Fee-For-Service claims database (2007-2018)	(1) 365-day of continuous enrolment in Medicare parts A, B and D before cohort entry (2) Patients required to have ≥2 claims with PAH diagnosis during baseline period	Sildenafil Tadalafil	Endothelin receptor antagonist	Total: 5,776 Treatment: 2,888 Control: 2,888	3989 (69.1%)	74 (range: 65-96)	Total: 114 Treatment: 55 Control: 59	Treatment: 168 days (37-530) Control: 151 days (47-508)
Huo, 2023^a,d^	Retrospective Case Control	USA: IBM, R MarketScan, R Medicare Supplemental Database (2016 to 2019)	(1) Continuous insurance coverage from 2016 to 2019 (2) >65 years old at start of study (3) AD diagnosis date should be after the date sildenafil is prescribed	Sildenafil	Non-use	Total: 27,174 Treatment:13,587 Control: 13,587	364 (0.01%)	NA	Total: 2071 Treatment: 775 Control: 1296	Treatment: 4 years Control: 4 years
Braun, 2023^a,b^	Retrospective Cohort Study	USA: Cancer of the Prostatic Strategic Urologic Research Endeavor registry (CaPSURE) (1998-2022)	(1) Men aged ≥40 years with a new diagnosis of ED between January 1, 2000, and March 31, 2017.	Sildenafil	Non-use	Total: 5,937 Treatment: 3,161 Control: 2,776	0 (0%)	66 (IQR: 60-72)	Total: 248 Treatment: 98 Control: 150	Treatment: 140 months Control: 124 months
Adesuyan, 2024^c^	Retrospective Cohort Study	UK: IQVIA Medical Research Data (2000-2017)	(1) At least 50 years of age at time of PCa diagnosis who underwent primary management (2) At least 5 years of post-treatment follow up.	Sildenafil Tadalafil Vardenafil	Non-use	Total: 269,725 Treatment: 147,989 Control: 121,736	0 (%)	58 (10)	Total: 1119 Treatment: 749 Control: 370	Total: 5.1 years (2.9-8.9)

NA: Not applicable.

^a^Did not include interquartile range (IQR) for duration of follow up.

^b^Measured Alzheimer Disease and related dementia.

^c^Did not include duration of follow-up for treatment and control group.

^d^Did not include mean/median data. 19073 (70.2%) were 65-74 and 8101 (29.8%) were ≥75 years old.

Of the 5 studies involving 885,380 patients, 284,037 patients were in the treatment group and 601,343 were in the control group. The occurrence of AD in the treatment and control group were pooled using the random-effects model. Adesuyan et al. and Braun et al. did not include any females, while Huo et al., Fang et al. and Desai et al. included study populations that comprise of 0.01%, 2.0% and 69.1% females respectively. Using the Newcastle-Ottawa Scale, all of the studies included are of good quality (Supplementary Table 2).

### Primary outcome: Alzheimer disease

### Sildenafil

**Figure 2:**
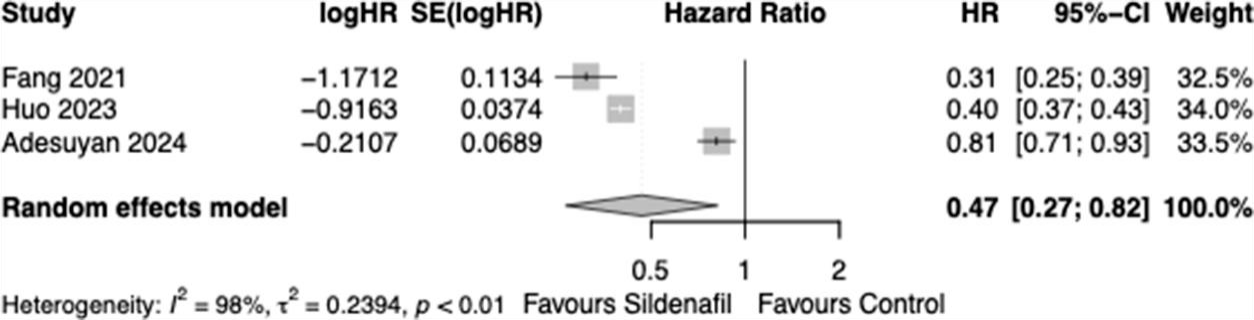
Forest plot of hazard ratio (HR) of patients developing Alzheimer disease in Sildenafil vs. control group.

HR of AD in patients on Sildenafil compared to non-use was pooled across 3 studies and the difference between the groups was significant (HR: 0.47, 95% CI: 0.27-0.82, p<0.001), with an I^2^ index of 98% (Figure 2). The 3 studies involved 873,667 patients, 277,988 patients were in the treatment group and 595,679 were in the control group. Influence analysis conducted revealed no outliers (Supplementary Figures 1, 2).

### PDE5 inhibitors

**Figure 3:**
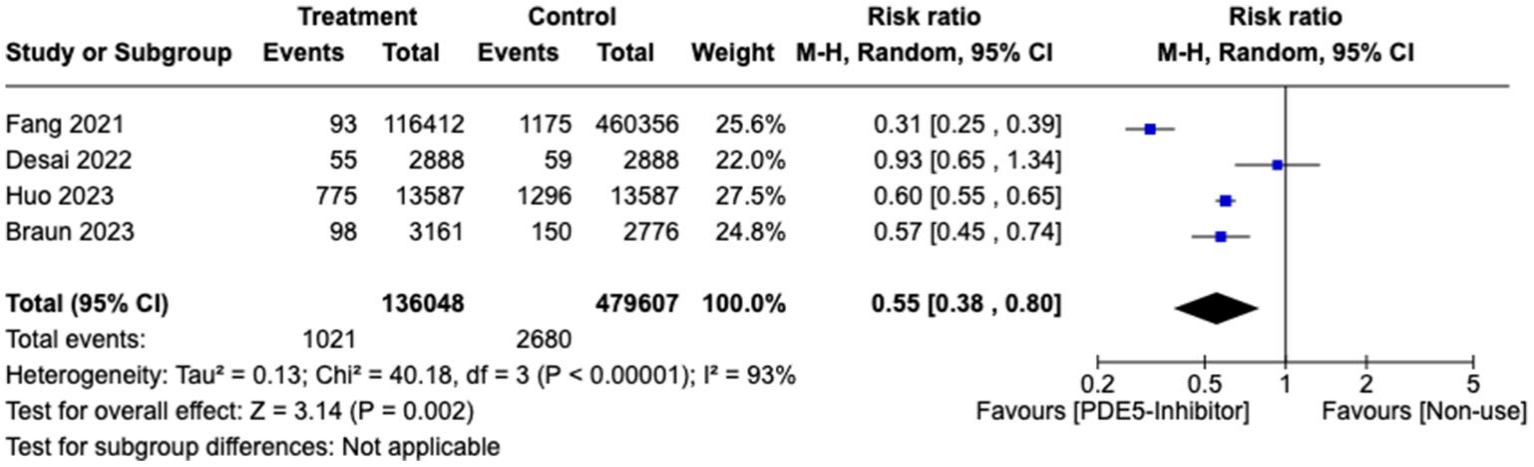
Forest plot of risk ratio (RR) of patients developing Alzheimer disease in treatment vs. control group.

Initially, 5 studies involving 885,380 patients (284,037 and 601,343 patients in the treatment and control group respectively) were pooled and we found that patients on PDE5 inhibitors were not at significantly lower risk of developing AD compared to controls (RR: 0.70, 95% CI: 0.32-1.52, p<0.01), with an I^2^ index was 98%. Influence analysis revealed 1 outlier, Adesuyan et al. [12] and a sensitivity analysis excluding it was conducted (Supplementary Figures 3, 4). The pooled risk ratios of the remaining 4 studies revealed significant lower reduction of AD in patients on PDE5 inhibitors (RR: 0.55, 95% CI: 0.38-0.80, p=0.002) compared to non-use, with an I^2^ index of 98.0% (Figure 3). The 4 studies involved 615,655 patients, 136,048 patients were in the treatment group and 479,607 were in the control group.

### Subgroup analysis: gender

**Figure 4:**
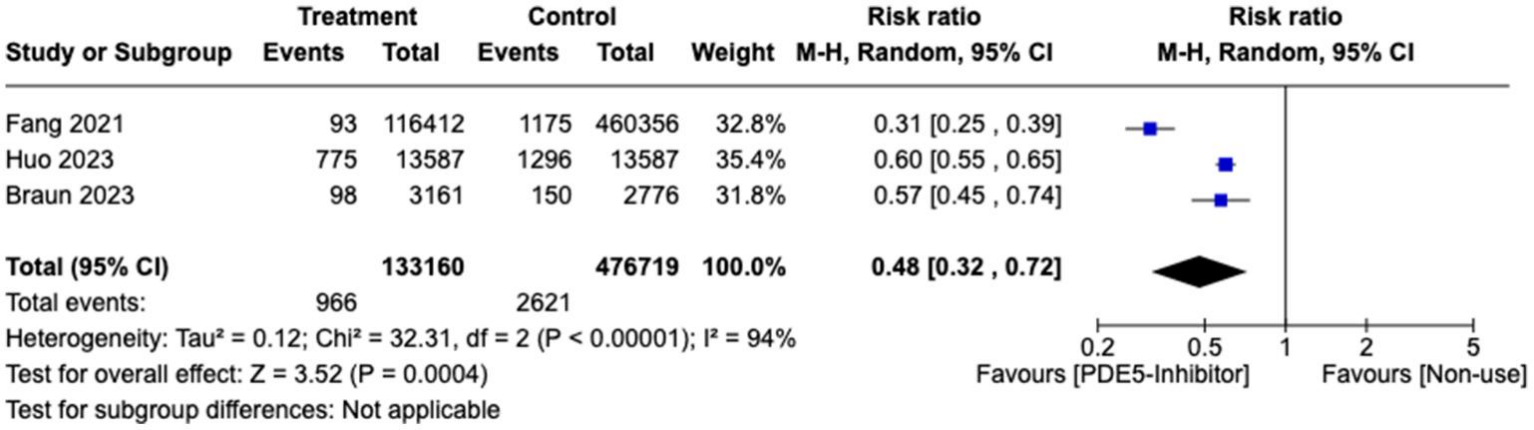
Forest plot of subgroup analysis of risk ratio (RR) of male patients developing Alzheimer disease in treatment vs. control group.

Fang et al., Huo et al., Braun et al. and Adesuyan et al. included 2.0%, 0.01%, 0% and 0% females respectively. Initially, 4 studies involving 879,604 patients (281,149 and 598,455 patients in the treatment and control group respectively) were pooled and we found that patients on PDE5 inhibitors were not at significantly lower risk of developing AD compared to controls (RR: 0.65, 95% CI: 0.22-1.97, p<0.01), with an I^2^ index was 99%. Influence analysis revealed 1 outlier, Adesuyan et al. [12] and a sensitivity analysis excluding it was conducted (Supplementary Figures 5, 6). Subgroup analysis by gender with the remaining 3 studies found that a significant reduction of AD in patients on PDE5 inhibitors (RR: 0.48, 95% CI: 0.32-0.72, p=0.002) compared to non-use in males (Figure 4). The 3 studies involved 609,879 patients, 133,160 patients were in the treatment group and 476,719 were in the control group.

## DISCUSSION

To address the association between the use of Sildenafil and risk of AD, we conducted a systematic review and meta-analysis involving 885,380 patients, to determine if Sildenafil use is associated with reducing the risk of developing AD. We found that Sildenafil was associated with a two-fold reduction in AD (HR: 0.47, 95% CI: 0.27-0.82, p<0.001) compared to non-use. In addition, when other formulations of PDE5 inhibitors were included, it was associated with a significant reduction in AD (RR: 0.55, 95% CI: 0.38-0.80, p=0.002) compared to non-use.

The cause of the disparity in findings among the individual studies may be due to different study population, control group, sample size and duration of follow-up. Desai et al. compared PDE5 inhibitors (Sildenafil/Tadalafil) against Endothelin receptor antagonists (ERA) in a cohort with pulmonary hypertension while other studies included in our meta-analysis compared between use of Sildenafil vs. non-use [11, 12, 16, 17]. Furthermore, the sample size in Desai et al. was relatively small. In comparison to Adesuyan et al. [12] which studied 269,725 patients, Desai et al. included 5776 patients [10]. In addition, AD is an age-dependent chronic disease and in comparison to the median follow-up duration of 132 months in Braun et al., [16] the median follow-up of 151-168 days in Desai et al. may have caused their study to be underpowered and AD to be underrepresented [10, 18].

In total, 1770 patients (0.62%) in the treatment group developed AD while 3050 patients (0.51%) in the control group developed AD. While this is inconsistent with the findings of our study, the difference in incidence can be explained by the disparity in Adesuyan 2024 where the treatment (0.51%) and control (0.30%) group had 749 and 370 AD diagnosis over 925,969 and 383,236 person years respectively [12]. In addition, based on our influence analysis using RRs of studies, we identified Adeyusan et al. as a potential outlier. The difference in study duration would potentially create a confounder as a study duration would allow a greater incidence of AD to occur. To mitigate this confounder, Adeyusan et al. utilized hazard ratios to identify the risk of developing AD in patients on treatment vs. control. Hazard ratios measure the rate of developing Alzheimer Disease over time and summarize the treatment effect over the study duration [19, 20]. Furthermore, the incidence ratios of Fang 2021, Desai 2022, Huo 2023 and Braun 2023 consistently showed that the control arm had a higher incidence of AD over time than the treatment arm (2.55% vs. 0.08%, 2.04% vs. 1.90%, 9.54% vs. 5.70% and 5.40% vs. 3.10%) respectively [10, 11, 16, 17].

A recent meta-analysis by Abouelmagd et al. similarly concluded that the use of PDE5 inhibitors is associated with a reduced risk of AD [21]. However, the methodology of our study differs from Abouelmagd et al. Firstly, our study differs in terms of the quality and characteristics of studies included. In our study, we presented in detail the study characteristics as well as the control group (Table 1). While Abouelmagd et al. included Wilkinson et al., the characteristics of the study were misrepresented and there was no mention of control groups [22]. In Wilkinson et al., the authors analysed the risk of dementia in patients with 744 different medications. Furthermore, while the study included 551,344 patients, only 1142, 230 and 466 patients were on any PDE-5 inhibitors such as Sildenafil, Vardenafil and Tadalafil respectively [22]. However, Abouelmagd et al. quoted that almost a third of medications were associated with dementia without mentioning the exact number of patients on PDE-5 inhibitors [21]. Secondly, in our analysis, we included HRs that had been provided by the respective studies. However, in Abouelmagd et al., there are discrepancies in the HRs ratios included in the analysis and no mention of the methodology in deriving the hazard ratios. In Henry et al., the results were in odds ratio and Abouelmagd et al. converted it to HR in the meta-analysis [23]. Furthermore, in Adesuyan et al., while the final HR was 0.82 (0.72-0.93), a subgroup analysis finding of HR: 0.65 (0.49-0.87) was selected without justification, potentially skewing the results [12]. Therefore, our study differs from Abouelmagd et al. in terms of the quality of studies included and the accuracy of the meta-analysis.

The benefit of PDE5 inhibitors in AD has been demonstrated in mouse model studies where Sildenafil has been shown to improve the learning and memory abilities in mouse with learning impairments [9] and restore cognitive function in mouse with AD [24]. The use of Sildenafil also reduces the levels of amyloid-β (Aβ) peptide, a hallmark of AD, in the hippocampus of mouse models [25, 26]. Evaluation of multiple pathways linked to the activity of Sildenafil has uncovered the nitric oxide synthase/nitric oxide/cyclic guanosine monophosphate (NOS/NO/cGMP) signalling pathway to be a key pathway [25, 27]. In AD, the cGMP signalling is compromised and PDE5, which degrades cGMP, is upregulated. As a PDE5 inhibitor, Sildenafil inhibits PDE5 and increases cGMP levels. The increased cGMP levels activate peroxisome proliferator-activated receptor-γ coactivator 1α (PGC1α) which induces mitochondrial biogenesis and anti-oxidant enzymatic action [28, 29]. Furthermore, Sildenafil promotes smooth muscle relaxation and vasodilation via the cGMP pathway, improving cerebral blood flow and reducing hippocampus hypoperfusion [30, 31]. While animal models demonstrated the efficacy of other forms of PDE5 inhibitors, only 2 studies in our meta-analysis investigated other PDE5 inhibitors.

AD is the most common cause of dementia, accounting for 60% to 80% of patients. In our meta-analysis, three studies using Sildenafil focused exclusively on AD [11, 12, 17]. Although our study found that the use of Sildenafil reduces the risk of AD, it is unclear whether Sildenafil could potentially reduce the risk of developing other subtypes of dementia. Animal data by Vaskat et al. found that the use of Sildenafil in mouse with vascular dementia led to improved cognition and memory [32]. Sildenafil increases cGMP and enhances nitric oxide-mediated vasodilation and cerebral blood flow [33]. In mouse with ischemic stroke, Sildenafil significantly promotes neurogenesis and improves neurological functional outcome [34]. While the potential of Sildenafil on animal models is promising, further clinical and experimental studies are required to examine the role of Sildenafil in other subtypes of dementia.

Although studies have shown that females are at greater risk of developing AD, [35, 36] the proportion of females included our studies are exceedingly low, ranging from 0% to 0.02%, with the exception of Desai et al. which had 69.1% females [10–12, 16, 17]. Furthermore, females accounted for less than 0.2% in the 3 studies involved in the pooled HR ratios of Sildenafil [11, 12, 17]. Future studies should consider increasing female representation given the inherent gender risk of females.

Our study has some inherent limitations. First, our meta-analysis was unable to examine the association of Sildenafil use and other subtypes of dementia. Second, all the studies conducted were conducted on retrospective datasets. As a result, the duration and details on the treatment regimens of the patients and compliance were not available. Third, selection bias cannot be excluded and some patients may be lost to follow-up. Last, as expected, the majority of these studies involved less than 0.2% of females, making it difficult to extend our findings to the female population.

Future double blind randomized control trials using a standardised diagnostic and monitoring protocol with correlation of imaging and biological markers will provide new insights. The dosage effect of Sildenafil on the clinical and amyloid load on neuroimaging can also be further examined.

## CONCLUSIONS

Our meta-analysis showed that the use of Sildenafil is associated with a reduced risk of developing AD by about two-fold. Further randomized control trials to ascertain if Sildenafil use can reduce amyloid load in the brain will provide more conclusive evidence supporting its neuroprotective effect.

## Supplementary Material

Supplementary Figures

Supplementary Tables
