# Supplementary material for: Sildenafil and risk of Alzheimer disease: a systematic review and meta-analysis
Source: Aging (Albany NY). 2025 Mar 17;17(3):726–39. doi: 10.18632/aging.206222 (PMC11984433; doi:10.18632/aging.206222)
Supplement: Supplementary Figures [file aging-17-206222-s001.pdf]

SUPPLEMENTARY FIGURES

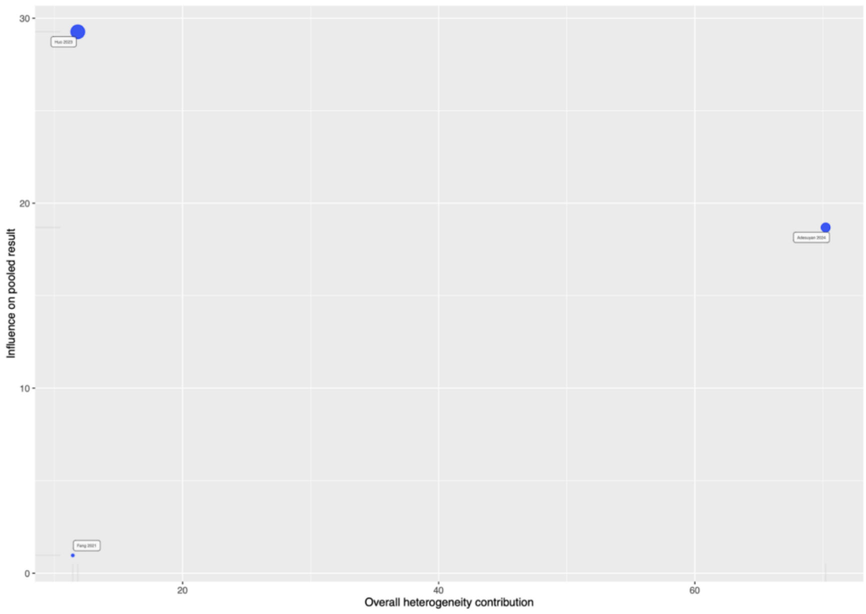

Supplementary Figure 1. Baujat plot on studies for hazard ratio (HR) of patients developing Alzheimer disease in Sildenafil vs. control group.

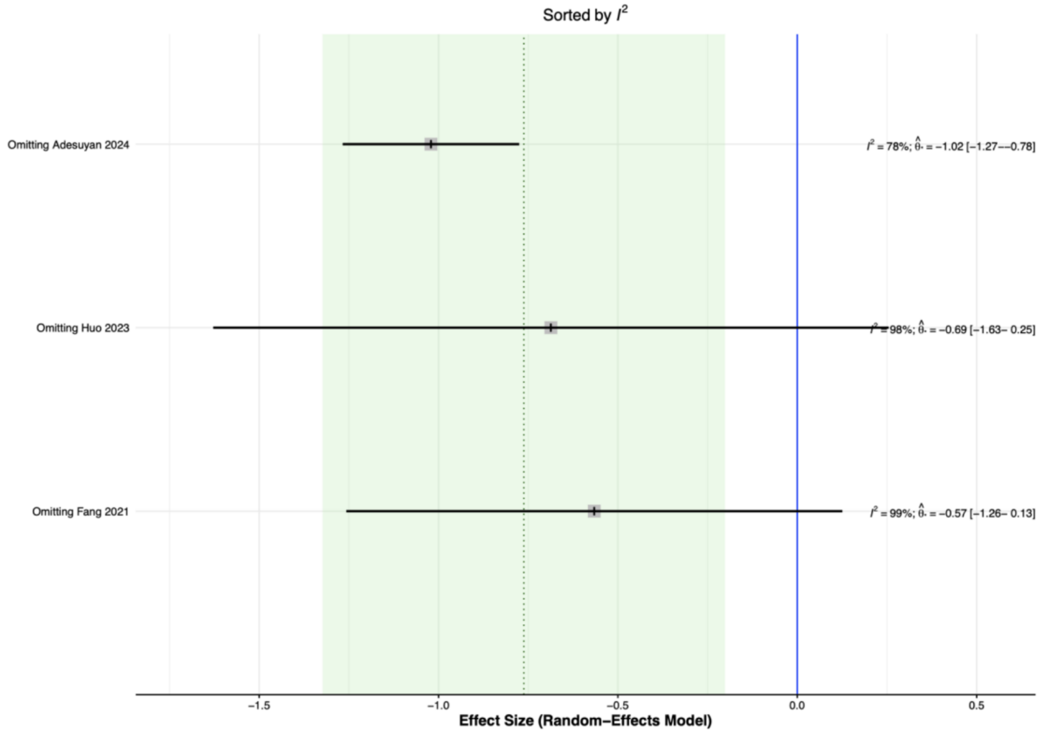

Supplementary Figure 2. Leave one out analysis on studies for hazard ratio (HR) of patients developing Alzheimer disease in Sildenafil vs. control group.

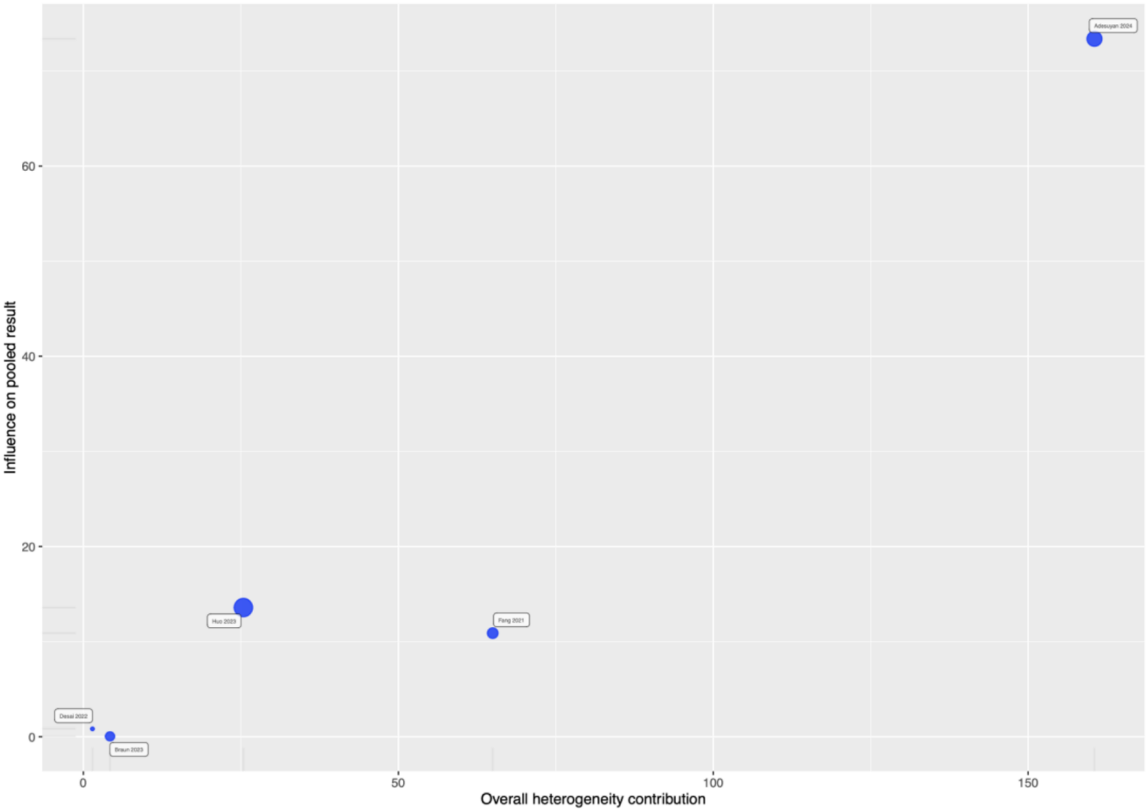

Supplementary Figure 3. Baujat plot on studies for risk ratio (RR) of patients developing Alzheimer disease in treatment vs. control group.

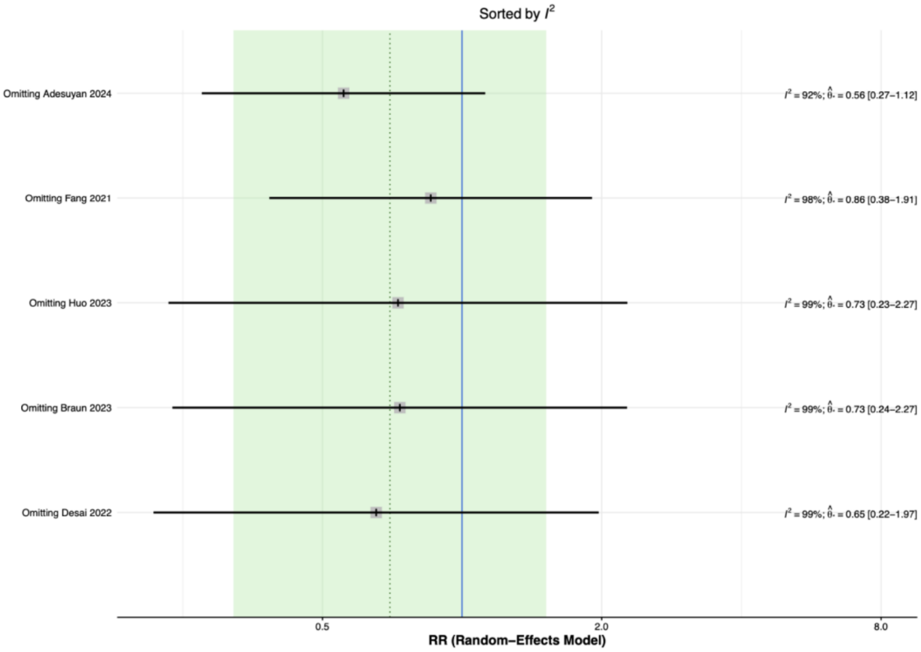

Supplementary Figure 4. Leave one out analysis on studies for risk ratio (RR) of patients developing Alzheimer disease in treatment vs. control group.

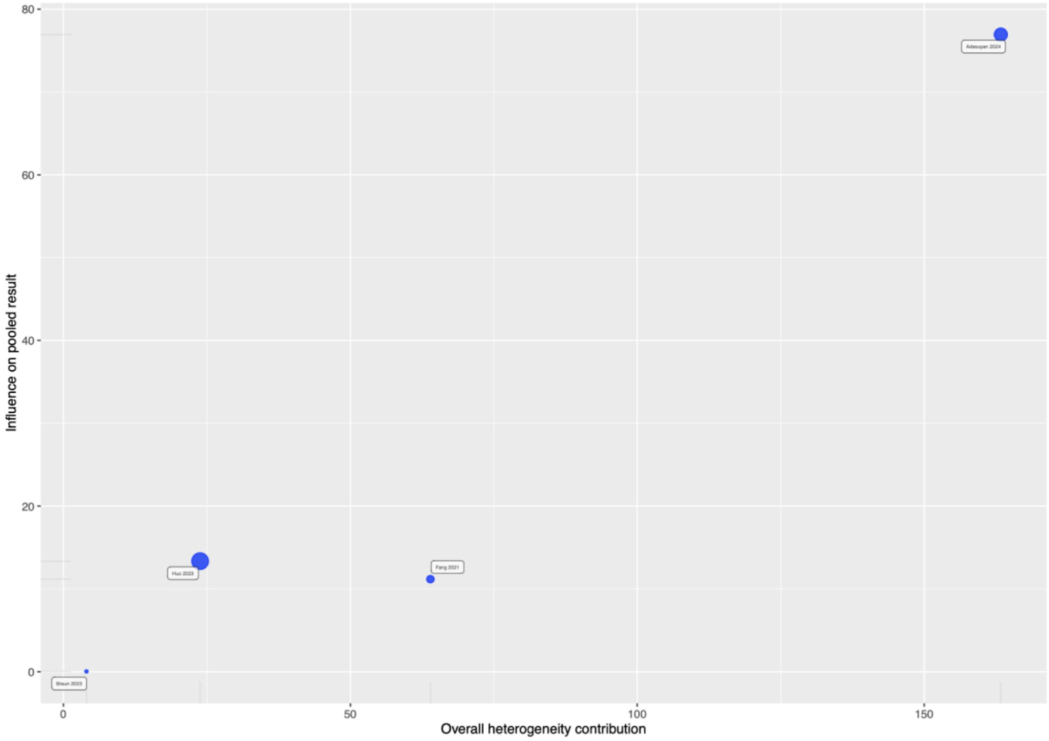

Supplementary Figure 5. Baujat plot on studies for risk ratio (RR) of male patients developing Alzheimer disease in treatment vs. control group.

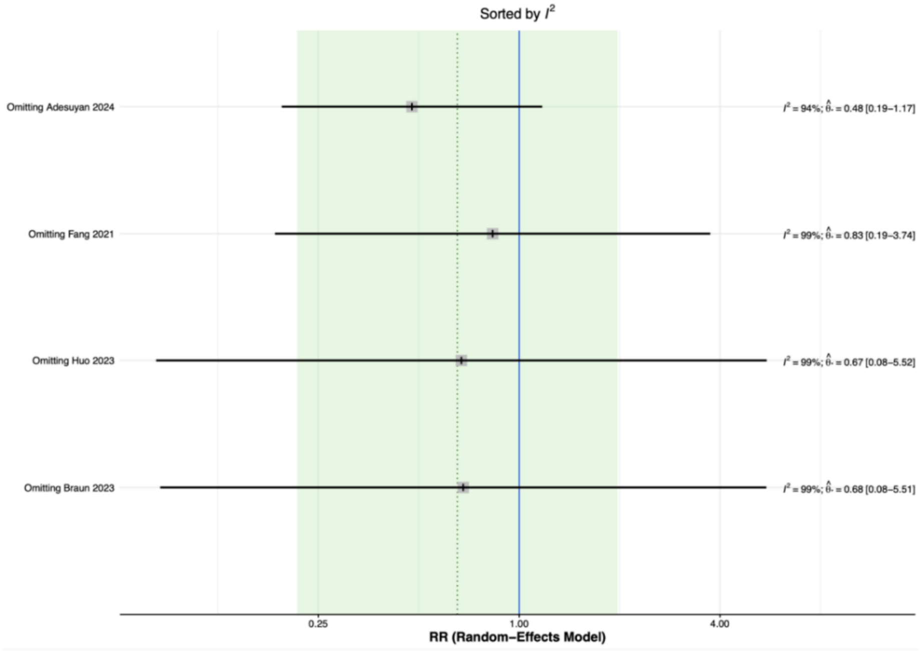

Supplementary Figure 6. Leave one out analysis on studies for risk ratio (RR) of male patients developing Alzheimer disease in treatment vs. control group.
