# Supplementary material for: Sildenafil and risk of Alzheimer disease: a systematic review and meta-analysis
Source: Aging (Albany NY). 2025 Mar 17;17(3):726–39. doi: 10.18632/aging.206222 (PMC11984433; doi:10.18632/aging.206222)
Supplement: Supplementary Tables [file aging-17-206222-s002.pdf]

SUPPLEMENTARY TABLES

Supplementary Table 1. Search strategy.

MEDLINE

As of March 11 2024.

	Search terms	Hits
1	exp sildenafil/ or sildenafil*.ab,ti.	8642
2	exp viagra/ or viagra*.ab,ti.	6278
3	exp phosphodiesterase 5 inhibitor/ or phosphodiesterase 5 inhibitor*.ab,ti. or phosphodiesterase type 5 inhibitor*.ab,ti. or PDE5I*.ab,ti. or PDE5 inhibitor*.ab,ti.	11490
4	exp alzheimer disease / OR alzheimer*.ab,ti.	205190
5	1 OR 2 OR 3	13574
6	4 AND 5	96

Embase

As of March 11 2024.

	Search terms	Hits
1	'sildenafil'/exp OR ‘viagra’/exp OR ‘sildenafil citrate’/exp OR ‘phosphodiesterase 5 inhibitor*’/exp OR ‘PDE5I*’/exp OR ‘PDE5 inhibitor*’/exp	29862
2	'sildenafil':ab,ti OR ‘viagra’:ab,ti OR ‘sildenafil citrate’:ab,ti OR ‘phosphodiesterase 5 inhibitor*’:ab,ti OR ‘phosphodiesterase type 5 inhibitor*’:ab,ti OR ‘PDE5I*’:ab,ti OR ‘PDE5 inhibitor*’:ab,ti	18512
3	'alzheimer disease'/exp	257057
4	‘alzheimer disease’:ab,ti,kw OR ‘alzheimer’:ab,ti,kw	267179
5	#1 OR #2	31248
6	#3 OR #4	314532
7	#5 AND #6	319

Supplementary Table 2. Quality assessment of 5 studies using Newcastle-Ottawa scale.

Author	Study design	Newcastle-Ottawa scale		
		Selection	Comparability	Exposure
Fang, 2021	Case Control	★★★	★★	★★★
Huo, 2023	Case Control	★★★	★★	★★★
Desai, 2022	Cohort Study	★★★★	★★	★★★
Braun, 2023	Cohort Study	★★★★	★★	★★
Adesuyan, 2024	Cohort Study	★★★★	★★	★★★
